# Own-gender bias in facial feature recognition yields sex differences in holistic face processing

**DOI:** 10.1186/s13293-025-00695-7

**Published:** 2025-02-19

**Authors:** Tobias Hausinger, Björn Probst, Stefan Hawelka, Belinda Pletzer

**Affiliations:** 1 Centre for Cognitive Neuroscience, University of Salzburg, 5020 Salzburg, Austria; 2 Department of Psychology, University of Salzburg, 5020 Salzburg, Austria

## Abstract

**Introduction:**

Female observers in their luteal cycle phase exhibit a bias towards a detail-oriented rather than global visuospatial processing style that is well-documented across cognitive domains such as pattern recognition, navigation, and object location memory. Holistic face processing involves an integration of global patterns and local parts into a cohesive percept and might thus be susceptible to the influence of sex and cycle-related processing styles. This study aims to investigate potential sex differences in the part-whole effect as a measure a of holistic face processing and explores possible relationships with sex hormone levels.

**Methods:**

147 participants (74 male, 51 luteal, 22 non-luteal) performed a part-whole face recognition task while being controlled for cycle phase and sex hormone status. Eye tracking was used for fixation control and recording of fixation patterns.

**Results:**

We found significant sex differences in the part-whole effect between male and luteal phase female participants. In particular, this sex difference was based on luteal phase participants exhibiting higher face part recognition accuracy than male participants. This advantage was exclusively observed for stimulus faces of women. Exploratory analyses further suggest a similar advantage of luteal compared to non-luteal participants, but no significant difference between non-luteal and male participants. Furthermore, testosterone emerged as a possible mediator for the observed sex differences.

**Conclusion:**

Our results suggest a possible modulation of face encoding and/or recognition by sex and hormone status. Moreover, the established own-gender bias in face recognition, that is, female advantage in recognition of faces of the same gender might be based on more accurate representations of face-parts.

**Supplementary Information:**

The online version contains supplementary material available at 10.1186/s13293-025-00695-7.

## Abstract (plain language summary)

In this study, participants were required to recognize a previously encountered face from one of two options. The correct face and the distractor face did only differ in one certain face part, that is, either the eyes, nose or mouth. When participants were presented only with the respective face parts instead of complete faces, female participants during their luteal cycle phase were more accurate in recognizing these parts than male participants. This advantage was observed only if female participants had to recognize face parts of women. Since previous studies have shown a female advantage in utilizing detail information, for instance when having to process local features within a global pattern or memorizing the location of features on a map, our findings represent a good fit with existing literature. Moreover, previous findings of better female recognition of women’s faces may be attributed to enhanced memory for individual face parts.

**Supplementary Information:**

The online version contains supplementary material available at 10.1186/s13293-025-00695-7.

## Highlights

- Luteal phase female participants show a significantly smaller part-whole effect than male participants.
- Luteal phase female participants were significantly more accurate in recognizing face-parts of women than male participants.
- Testosterone partially mediates the observed sex difference in the part-whole effect.

**Supplementary Information:**

The online version contains supplementary material available at 10.1186/s13293-025-00695-7.

## Introduction

Faces are considered as one of the most, if not the most, important visual cues in social interaction. Human proficiency in face processing is remarkable, permitting fast and precise recognition under challenging viewing conditions and across a wide range of viewing angles [1]. While regular objects are predominantly processed in a feature-based manner, especially when they are unfamiliar or viewed from an unusual perspective, face processing is assumed to involve a comparatively high degree of holistic processing [2]. Accordingly, face recognition relies on an efficient synergy of both the particular attributes of local facial features and their global spatial configuration. Despite a lack of consensus on specific functionality, holistic face processing is considered adaptive for various aspects of identity detection, expression categorization, and social perception (see [3] for a review).

Sex differences in face recognition are well-documented, with female observers, on average, outperforming male observers in identifying unfamiliar faces with a neutral facial expression [4]. In particular, female observers commonly show a so-called “own-gender bias”, that is, higher accuracy in identifying faces of women than faces of men [4]. Correspondingly, in female observers, the Fusiform Face Area (FFA), a region within the mediolateral fusiform gyrus specialized in face-processing [5, 6], displays greater activation for faces of women than faces of men [7]. Moreover, female observers demonstrate advantages in recognizing and distinguishing emotional expressions [4, 8], including a comparatively higher effective connectivity from the FFA to the limbic system [4]. However, while behavioral and structural sex differences related to face recognition are well-documented though not without controversy [9, 10], established evidence on sex and sex hormone related differences in visuospatial processing styles may provide a closer look into some of the underlying mechanisms. Since these differences range from detail-oriented to global visuospatial processing biases, an impact of sex and sex hormones on encoding and retrieval of facial features and their spatial configuration seems highly plausible and calls for further investigation. Sex differences in global–local processing styles range from basic hierarchical letter or shape tasks to more complex three dimensional environments. Therefore, the underlying tasks follow the tradition of classic global–local processing paradigms, as both levels involve a distinction that is purely spatial. This means that the global and local aspects of these stimuli differ in size and position. However, the global level does not represent a dominant Gestalt form that must first be broken down, as is often the case with traditional embedded figures tasks, where observers must recognize geometric shapes within a larger, superordinate object. Moreover, these global–local tasks typically involve hemispheric lateralization, with its direction and magnitude highly dependent on task and stimulus specific characteristics.

On average, female observers display a more local or detail-oriented processing style than male observers, a potential trait that is reflected in faster reaction times for local feature recognition [11–14] and greater dependence on local cues, that is, concrete landmarks, during spatial navigation [15–17]. Moreover, a female advantage in spatial object location memory tasks (see [18] for a review) suggests comparatively more detailed female spatial memory representations. Female reliance on local or detail-oriented processing styles further revealed substantial variation over the course of the menstrual cycle, with the most reliable evidence for a female local processing advantage emerging during the high estradiol and progesterone luteal phase. Sex differences in visuospatial processing styles are also supported by eye-tracking results indicating longer and more frequent fixations on local elements of a visual stimulus as well as increased focus on detail landmark information in female compared to male participants [19, 20], but see [21]. Considering these findings, it is yet to be determined whether sex-related or hormone-influenced differences in visuospatial processing may contribute to sex differences in holistic face encoding and recognition.

Perspectives on the nature of holistic face processing differ, with the core of the discussion revolving around whether such processing is Gestalt-like, that is, intrinsically more than the sum of individual parts by combining configural and featural information, or primarily dependent on the spacing between features, regardless of their particular shape [22]. As a result, available paradigms like the part-whole or face composite task do not share a common theoretical construct and are thus likely to provide empirical evidence for different aspects of holistic face processing [2, 23, 24]. The part-whole task, also used in this study, investigates holistic face processing by presenting participants with a target face followed by a recognition test. During this test, participants must either identify the complete target face or a single facial feature against a distractor. Assuming that recognition accuracy for individual facial features will be enhanced when these features are contextualized within the full facial structure rather than presented in isolation, the resulting part-whole effect is predicated on a gestaltist perspective of holistic face processing.

It should be noted that a global bias in pattern recognition or object location memory tasks indicates a preference for configural processing over featural details, which differs from the gestaltist integration associated with holistic processing. For instance, a local or detail-oriented approach, which is posited to be more pronounced in luteal phase observers, would predict a higher accuracy in recognizing isolated face parts and, as a consequence, a reduced part-whole effect. Considering holistic representations as a combination of configural and featural information, we would expect face representations of luteal phase observers to contain richer featural information, similar to findings of richer landmark- or location-specific object information within environmental representations of female observers [18].

While sparse available evidence does not support sex differences in holistic face processing [25], there might be some limitations due to a lack of hormonal status control and task related methodology. More specifically, sex differences in holistic processing had been assessed through memory differences between upright and inverted faces, an approach that might not exhibit the required selectivity between holistic and detail-oriented processing aspects and has been met with major concern [26]. In addition, face gender, a crucial factor in female face recognition advantage and presumably an important influence on the formation of face representations, has not yet been considered. Thus, to the best of our knowledge, the current study is the first to investigate sex- and sex hormone- dependent differences in holistic face processing by using the part-whole task, a paradigm considered to yield a purer measure of holistic face processing than regular face inversion tasks. Furthermore, we evaluate the importance of the gender of face stimuli and present the first investigation of holistic face processing that is also controlling for sex hormone levels, menstrual cycle and other modulating factors of hormonal status such as oral contraceptive (OC) use and endocrinological disorders.

Finally, sex differences in overt visuospatial attention as indicated by eye fixations have previously been related to performance differences in global–local processing studies [19, 20]. However, other studies have found performance differences without significant sex differences in fixations, suggesting that observers may just differ in how they weigh and encode identical information [27]. Since the recognition phase of the current experiment does not impose time constraints on participants, enabling natural scanning behavior, we would also like to take this opportunity to explore potential sex differences in eye fixation patterns.

## Methods

### Participants

159 participants (84 female, 75 male) were recruited for the current study. Participants were included if they were between 18 and 35 years old, right-handed and did not suffer from a psychological, endocrinological or neurological disorder according to self-reports. Since previous studies demonstrate potential effects of hormonal contraceptive use and menstrual cycle phase on cognitive processing in general [28, 29] and holistic vs. decomposed processing strategies in particular [12–14] we included only female participants who had not used hormonal contraceptives for at least 6 months before participation and reported a regular menstrual cycle between 21 and 35 days [30].

Sample recruitment was geared towards 70% luteal cycle phase female participants and 30% non-luteal phase participants. This ratio allowed us to focus on the luteal group for the assessment of sex-differences, but also enabled us to conduct exploratory comparisons between luteal and non-luteal participants.

Female cycle phase was recorded based on self-reported menses onsets of the three previous cycle phases, next menses after participation (available for 33 participants) and salivary hormonal levels. Female participants were allocated to the luteal cycle phase, if participation was within 11–3 days before onset of the next menses and progesterone levels surpassed a threshold of 40 pg/ml. If the date of the next menses was not available, female participants were allocated to the luteal cycle phase if participation was after the expected date of ovulation calculated from the three previous cycles and confirmed by progesterone levels surpassing a threshold of 40 pg/ml (see supplementary material for an overview of all criteria, including allocation to non-luteal and anovulatory groups). Eleven female participants had to be excluded due to anovulatory cycles.

The remaining sample of 51 female luteal phase (mean cycle length: 29.07 days, SD = 2.48, cycle range 24.5–35 days; mean age = 23.47, SD = 3.31, age range 18–35 years), 22 female non-luteal phase (mean cycle length: 29.07 days, SD = 2.57, cycle range 25–33.5 days; mean age = 23.41, SD = 3.90, age range 18–32 years) and 74 male participants (mean age 23.65, SD = 2.99, range 18–33 years) were included in the statistical analysis. All participants were documented as cisgender, meaning that those assigned female at birth self-identified as women, and those assigned male at birth self-identified as men.

There were no significant age differences between groups F(2, 144) = 0.07, p = 0.932 and no significant IQ differences according to Raven’s APM Intelligence Screening between groups F(2, 144) = 1.10, p = 0.334.

Participants provided informed written consent before the experiment and were compensated with course credits or equivalent monetary payment. All methods conformed to the code of ethics constituted by the Declaration of Helsinki (2014).

### Stimuli and procedure

**Fig. 1:**
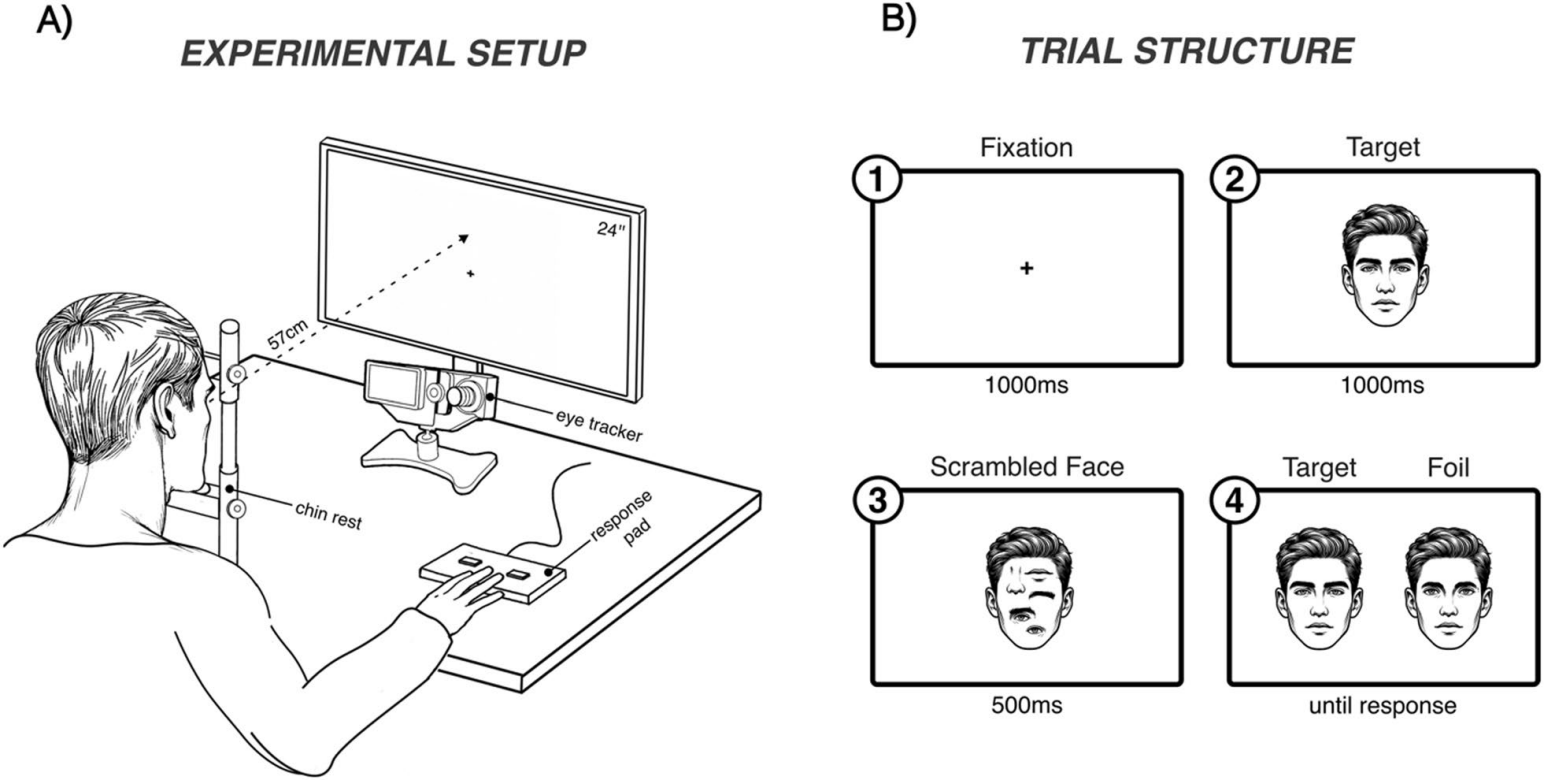
**A** Experimental Setup: Participants were seated in front of a 24-inch computer screen at a viewing distance of ~ 57 cm. Participant’s heads were stabilized by a forehead and chin rest. Target faces were indicated with a right-handed right or left button press. **B** Trial Structure: Each trial started with 1000 ms central fixation, followed by 1000 ms target presentation (study phase), 500 ms scrambled face mask presentation and ended with side-by-side target and foil presentation until response (forced-response phase). Note, that the actual experiment used photorealistic face stimuli (see Sect. "Stimuli and procedure".), and the displayed images are for illustrative purposes only

The overall procedure represents an adaptation of the original part-whole task by Tanaka and colleagues [31] and is illustrated in Fig. 1B.

Twenty Caucasian composite faces (ten female, ten male) were generated from digitally photographed grayscale faces included in the CAL/PAL Database [32]. All original photographs had to contain neutral face expressions. Composite faces were created for male and female faces separately and were based on Caucasian individuals. For each composite face, mouth, nose and eye features of three different original faces were placed inside identical face and hair outlines (templates). Eighteen composite faces (nine women, nine men) were used as whole face targets. Whole face foils were created by replacing only one critical face feature (mouth, nose or eyes) with a corresponding feature from other target faces of the same gender. Three part-face stimuli, depicting only isolated face features (mouth, nose or eyes) were created for each of the eighteen target faces and one part face stimulus was created for each of the 54 foil faces. Furthermore, one scrambled face mask was created for each face gender by randomly positioning the face features of two unrelated original photographs into the same face templates that were used for composite faces. In sum, 202 Stimuli were generated, with 18 whole face targets, 54 whole-face foils, 54 part-face targets, 54 part-face foils and 2 scrambled face masks.

Participants were seated in front of a 24-inch computer screen with a resolution of 1366 × 768 pixels and at a viewing distance of ~ 57 cm (Fig. 1A). Participant’s head positions were stabilized by a forehead and chin rest. Whole face stimuli (mid-forehead to chin x distance between ears) were presented at a size of 220 × 208 pixels (visual degrees: 8.61° × 8.03°), isolated mouth stimuli at 113 × 52 pixels (4.42° × 2.01°), isolated nose stimuli at 113 × 64 pixels (4.42° × 2.51°) and isolated eye stimuli at 181 × 61 pixels (7.02° × 2.41°). All stimuli were embedded in a 550 × 411 pixels white blank frame, with matching on-screen positions between part- and whole face stimuli. All pictures were created with Adobe Photoshop CC 2018 and exported to JPEG format before imported into the Eyelink1000 Experiment Builder software.

Each trial started with a central fixation cross for 1000 ms (Fig. 1B). Then, a whole study face was presented for 1000 ms, immediately followed by a 500 ms scrambled face mask. Next, target and foil stimuli were presented side by side and remained on-screen until a response was made. Participants indicated the target face using a button press (left or right) on a response pad with their right hand. Overall, the task included a total of 108 trials, starting with 12 training-trials from a randomly selected male and female face target followed by 96 test-trials using the remaining 18 face targets. Presentation order and target/foil-face position on-screen were pseudo-randomized and identical for every participant. Half of the trials contained a whole face target and a whole face foil varying in only one critical feature (mouth, nose or eyes), whereas the other half contained isolated face parts (targets and foil). For the inverted version of the part-whole task, all stimuli from the upright version were rotated 180°. Order of presentation was again pseudo-randomized and the same for each participant, but different from the upright condition. The inverted task was implemented as previous research has stated its necessity in controlling for encoding specificity effects during the upright part-whole task [33].

Upright and inverted part-whole tasks were the first two of four tasks within a more extensive test battery. Salivary samples were taken upon arrival and after the first, second and fourth task. A calibration sequence for the eye-tracking camera was carried out before each training and test phase. Once all four tasks were completed, subjects performed the Raven’s Progressive Matrices test for an intelligence screening plus a Premenstrual Syndrome-Questionnaire (exclusively for female participants). Overall, the whole experiment's mean duration was around 100 min, with five-minute breaks after the first, second and fourth task.

### Hormone level assessment

Progesterone, testosterone and estradiol levels were analyzed from passive drool method saliva samples using ELISA kits by DeMediTec. In order to average hormone levels over the entire course of our experiment, the four samples of the participant were pooled. Samples were stored at a temperature of − 20 °C and centrifuged twice at 3000 rpm for 15 and 10 min to remove any solid particles before analysis.

Estradiol, progesterone, and testosterone levels were compared between male, luteal phase female and non-luteal phase female participants using ANOVA and FDR corrected pairwise post-hoc comparisons. The analyses revealed a significant effect of group on progesterone F(2, 143) = 58.00, p < 0.001, testosterone F(2, 142) = 43.48, p < 0.001 and estradiol F(2,141) = 5.78, p = 0.004. Tukey’s HSD post-hoc tests revealed significantly higher progesterone levels in luteal female than in non-luteal female and male participants (all p_FDR_ < 0.001; compare Table 1), higher testosterone levels in male compared to luteal phase and non-luteal phase female participants (all p_FDR_ < 0.001) as well as higher estradiol levels in non-luteal phase female than luteal-phase female and male participants (all p_FDR_ < 0.005).

**Table 1:** Sex hormone levels in females and men

	*Estradiol *_*(n*=*144)*_	*Progesterone *_*(n*=*146)*_	*Testosterone *_*(n*=*145)*_
*♀luteal*	1.17 pg/mL ± 0.59	144.95 pg/mL ± 89.00***	63.13 pg/mL ± 33.41
*♀non-luteal*	1.67 pg/mL ± 0.76**	33.10 pg/mL ± 39.84	66.05 pg/mL ± 34.68
*♂male*	1.18 pg/mL ± 0.60	31.71 pg/mL ± 35.81	144.87 pg/mL ± 65.31***

Asterisks indicate sign. Differences to each of the other two groups (*p < 0.05, **p < 0.005, ***p < 0.001)

### Eye-tracking

Eye-tracking was performed with an EyeLink 1000 desktop mount system (SR Research, Ontario, Canada) at a sampling rate of 1,000 Hz. Calibration and validation were performed with a nine-point grid. For exploratory analyses of eye-fixations, only participants displaying an average validation error of 0.5° or less were included (M = 0.41°, SD = 0.07). To ensure constant eye tracking accuracy throughout the experiment, re-calibration was initiated whenever the eye tracking system did not detect a fixation on the fixation cross at the start of each trial. Only for the 54 whole face recognition trials, five areas of interest (AOI) were defined for each of the two faces during the recognition phase (right eye, left eye, center between eyes, lower third of the nose, mouth) and the combined number of fixations on both faces served as dependent variables in our models for each AOI respectively.

### Statistical analyses

Several linear mixed-effects models with random intercepts and fixed slopes were applied to the data using the *lmer-*function of the *lme4*-package [34] in R version 3.6.1. [35]. The models treated accuracy and reaction time (RT) as dependent variables and participant number (PNr) as random effect. For the investigation of sex differences, only female participants during the luteal cycle phase were included. Females during the non-luteal cycle phase were only included for exploratory analyses (Sect. "Including non-luteal cycle phase female participants"). Two baseline models were established: Variables part-whole condition (pw_condition), sex, and face gender, along with their interactions, were included as fixed effects [model (1): accuracy ~ 1 | PNr + pw_condition*facegender*sex; model (2): RT ~ 1 | PNr + pw_condition*facegender*sex]. Degrees of freedom were assessed using Satterthwaite’s approximation. FDR multiple comparison corrected P-values below 0.05 were considered statistically significant. For model (2), only correct trials and trials not deviating more than three standard deviations from the sample mean were included.

In addition, model (1) and (2) were computed as Bayesian Multilevel Regression models (BMLMs) using the Stan-based *brms* package [36, 37]. Assumptions regarding normality of residuals, normality of random effects, heteroscedasticity, collinearity and autocorrelations were confirmed for each baseline model using the *performance* package [38].

To assess mediating and moderating effects of sex hormones on sex differences in part-whole recognition accuracies, we conducted mediation analyses for each of the three sex hormones. In order to reduce data dimensionality, instead of adding pw_condition as fixed effect, a standardized part-whole effect (pwe) was calculated from the difference between part- and whole face response accuracies divided by the respective pooled standard deviations of the sample and used as dependent variable.

Mediation analyses included three models, testing (1) the relationship between sex and pwe [model (3): pwe ~ 1 | PNr + facegender*sex], (2) the relationship between sex and sex hormones [model (4): horm ~ sex], (3) and the relationship between sex and pwe with sex hormone as covariate [model (5): pwe ~ 1 | PNr + facegender*sex*horm]. Sex hormones were considered as mediators, if models (3) and (4) yielded significant effects and if there was a significant indirect effect of sex via sex hormone on pwe and if the direct effect of sex on pwe was reduced in model (5). In addition, if model (5) displayed a significant interaction between sex and sex hormones on pwe, sex hormones were considered as moderators. To examine sex differences in the number of eye fixations, Welch independent sample t-tests were calculated for each area of interest with sex as group factors [model (6): fixations_aoi ~ sex].

Since all hormone and fixation models were treated as exploratory analyses, no multiple comparison corrections were applied.

Data and scripts are made publicly available at: https://figshare.com/s/505b7fbd104b0cfe827a

## Results

### Task validation

As expected, we obtained a significant part-whole effect in accuracy measures for the upright face condition (β = 0.52, SE_β_ = 0.07, t(470) = 8.05, p < 0.001; *brms*: 95% CI [0.48, 0.80], PP_b > 0_ = 100%), but not for the inverted face condition (β = − 0.13, SE_β_ = 0.08, t(470) = − 1.73, p = 0.084; *brms*: 95% CI [− 0.38, 0.01], PP_b < 0_ = 96.67%) across the complete study sample. Thus, only when face representations were not affected by inverted presentation, participants recognized face parts more accurately in the context of a whole face than in isolation, suggesting the current task as a suitable adaptation for detecting the part-whole effect.

### Sex difference in holistic face processing

#### Accuracies

**Fig. 2:**
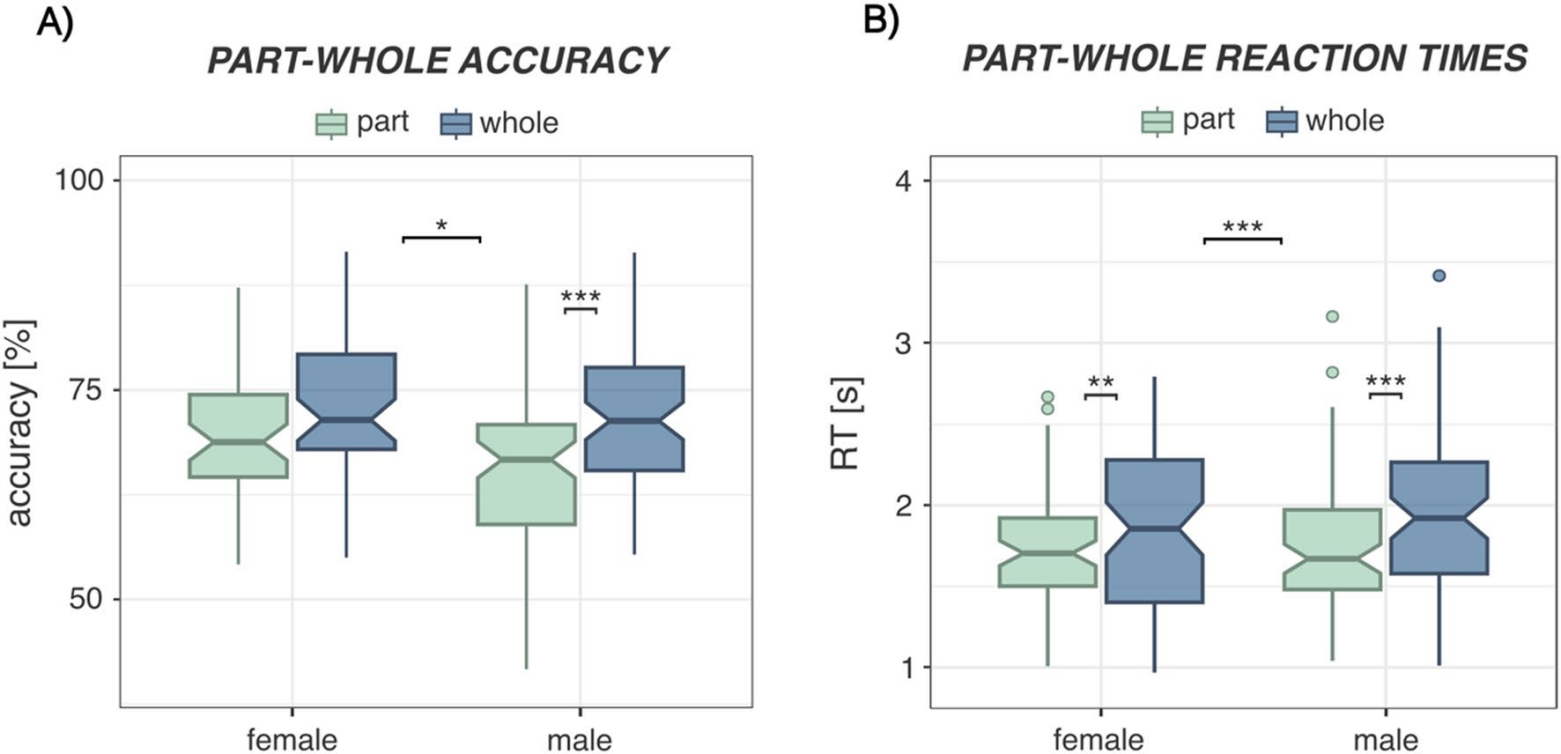
Sex differences in the part-whole effect. **A** Response accuracies: Female participants displayed a higher recognition accuracy in general. Male participants recognized significantly more whole faces compared to face parts, whereas no significant difference was present for female participants. **B** Reaction times: Male and female participants recognized face parts significantly faster compared to whole faces. This effect was significantly higher in male than in female participants. *p < 0.05, ** p < 0.01, ***p < 0.001. Notches represent the 95% CI for each median

There was a significant main effect of sex on response accuracy, suggesting more accurate face recognition in female than in male participants (compare Tables 2, 3). Also, there was a significant main effect of face gender on response accuracy, indicating that in general, faces of women were recognized more accurately than faces of men. Also, there were significant interactions of sex and part-whole condition (Fig. 2A) as well as sex and face gender on response accuracies. Finally, there was a moderate, non-significant interaction between face gender and part-whole condition as well as a moderate, non-significant three-way interaction between face gender, part-whole condition and sex.

**Table 2:** Results of Linear Mixed-Effects Models (LMM) and Bayesian Multilevel Regression Models (BMLM) for recognition accuracy

*Accuracy* ~ pw_condition*facegender*sex + *(1|PNr)*								
			LMM			BMLM		
	β	SE	df	t	p_FDR_	CI_low_	CI_up_	PP_b ≠ 0_ (%)
*(Intercept)*	0.23	0.13	400.90	1.70	0.089	− 0.02	0.49	96.30
*sex (male)*	− 0.66	0.17	400.90	− 3.81	< 0.001***	− 1.01	− 0.33	99.98
*pw_condition (whole)*	0.13	0.16	369.00	0.83	0.407	− 0.18	0.44	79.40
*facegender (man)*	− 0.51	0.16	369.00	− 3.18	0.003**	− 0.83	− 0.19	99.98
*sex: pw_condition*	0.49	0.21	369.00	2.32	0.021*	0.07	0.90	98.80
*sex: facegender*	0.56	0.21	369.00	2.73	0.013*	0.15	0.99	99.70
*pw_condition: facegender*	0.46	0.23	369.00	2.01	0.089	0.01	0.90	97.70
*sex: pw_condition: facegender*	− 0.50	0.30	369.00	− 1.68	0.104	− 1.05	0.09	95.60

*Standardized beta coefficients indicate changes in recognition accuracies when moving from level 0 to 1 (level 1 is indicated in brackets)*

**Table 3:** Mean accuracies (hP) and 95% confidence intervals (CI) for whole-face and face-part recognition for male, female luteal and female non-luteal participants

Accuracies							
		Male		Female luteal		Female non-luteal	
		Mean hP	CI 95%	Mean hP	CI 95%	Mean hP	CI 95%
Whole		0.72	[0.70, 0.74]	0.74	[0.71, 0.76]	0.76	[0.73, 0.79]
Part		0.66	[0.64, 0.67]	0.70	[0.68, 0.72]	0.69	[0.66, 0.73]

To resolve both significant interaction terms involving sex, we performed separate baseline models for male and female participants. While we obtained a significant part-whole effect in male participants irrespective of face gender (β = 0.61, SE_β_ = 0.13, t(219.00) = 4.64, p < 0.001), there was no significant part-whole effect in luteal phase female participants (β = 0.14, SE_β_ = 0.17, t(150.00) = 0.83, p = 0.410) but a significant interaction between face gender and part-whole condition (β = 0.48, SE_β_ = 0.24, t(150.00) = 2.01, p = 0.046). Accordingly, luteal phase participants displayed a significant part-whole effect for faces of women (β = 0.62, SE_β_ = 0.16, t(50.00) = 3.85, p < 0.001), but not for faces of men (β = 0.14, SE_β_ = 0.16, t(50.00) = 0.86, p = 0.392).

Irrespective of whether participants were responding to part or whole face stimuli, while luteal phase female participants recognized faces of women significantly more accurately than faces of men (β = − 0.54, SE_β_ = 0.17, t(150) = − 3.17, p = 0.002), there was no significant effect of face gender on recognition accuracy in male participants (β = 0.06, SE_β_ = 0.13, t(219) = 0.44, p = 0.662), supporting the notion of a female own-gender bias in face recognition within the current sample.

**Fig. 3:**
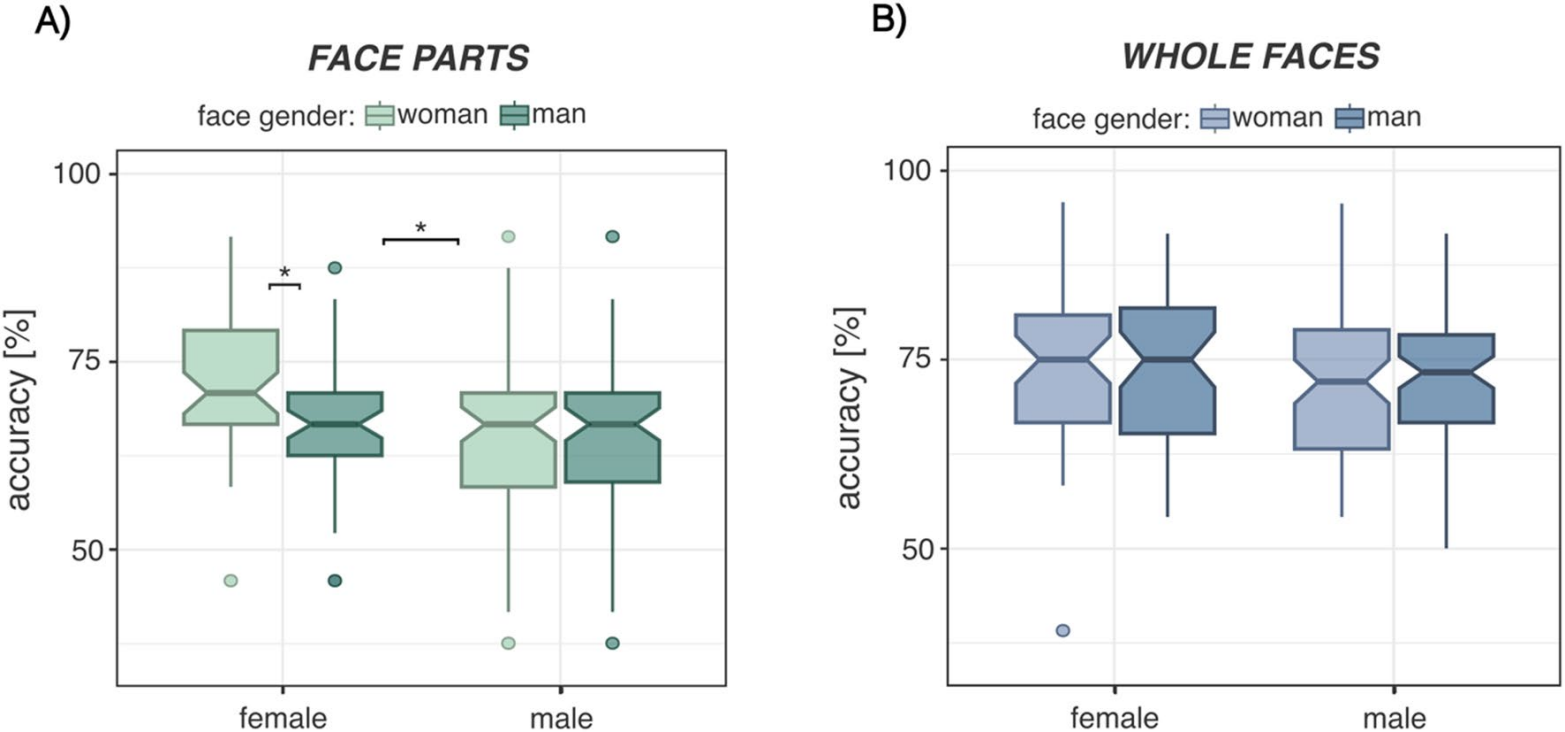
Effects of face gender on recognition accuracies in female and male participants for **A** face parts and **B** whole faces. While there was no difference in accuracies between faces of men and faces of women, female participants recognized significantly more face parts of women than face parts of men. Notches represent the 95% CI for each median

Moreover, this own-gender bias in luteal phase female participants was only significant for isolated representations of face parts (β = − 0.55, SE_β_ = 0.17, t(50) = − 3.20, p = 0.002), while it was not for whole faces (β = − 0.06, SE_β_ = 0.18, t(50) = − 0.31, p = 0.757) (Fig. 3).

There were no significant correlations between reaction times (RT) and accuracy (supplementary Table 2). When entering RT as a covariate to the model, discovered accuracy effects remained significant (supplementary Table 3).

#### Reaction times (RT)

There was a significant main effect of part-whole condition, indicating faster reaction times to face parts than whole faces (compare Tables 4, 5). Also, the part-whole effect in reaction times was significantly moderated by sex (Fig. 2B). Furthermore, there was moderate, non-significant interaction between sex and face gender on RT and a moderate, non-significant three-way interaction between sex, part-whole condition and face gender on RT. To resolve the significant interaction between part-whole condition and sex, we performed separate baseline models for male and female participants. Accordingly, there was a significantly stronger RT difference between face parts and whole faces in male (β = 0.58, SE_β_ = 0.08, t(219) = 7.21, p < 0.001) than in female participants (β = 0.33, SE_β_ = 0.09, t(150) = 3.57, p < 0.001).

**Table 4:** Results of Linear Mixed-Effects Models (LMM) and Bayesian Multilevel Regression Models (BMLM) for reaction times

*Reaction time* ~ pw_condition*facegender*sex + *(1|PNr)*								
			LMM			BMLM		
	β	SE	df	t	p_FDR_	CI_low_	CI_up_	PP_b ≠ 0_ (%)
*(Intercept)*	− 0.19	0.14	178.95	− 1.36	0.175	− 0.47	0.08	90.77
*sex (male)*	− 0.11	0.18	178.95	− 0.63	0.530	− 0.47	0.23	74.85
*pw_condition (whole)*	0.31	0.09	369.00	3.22	0.003**	0.12	0.50	99.90
*facegender (man)*	0.02	0.09	369.00	0.17	0.868	− 0.17	0.21	57.38
*sex: pw_condition*	0.30	0.12	369.00	2.41	0.021*	0.05	0.54	99.25
*sex: facegender*	0.20	0.12	369.00	1.59	0.113	− 0.04	0.44	94.15
*pw_condition: facegender*	− 0.04	0.13	369.00	− 0.30	0.762	− 0.30	0.22	62.58
*sex: pw_condition: facegender*	− 0.28	0.17	369.00	− 1.63	0.104	− 0.64	0.06	94.50

*Standardized beta coefficients indicate changes in reaction times when moving from level 0 to 1 (level 1 is indicated in brackets)*

**Table 5:** Mean reaction times (RT) and 95% confidence intervals (CI) for whole-face and face-part recognition for male, female luteal and female non-luteal participants

Reaction times						
	Male		Female luteal		Female non-luteal	
	Mean RT	CI 95%	Mean RT	CI 95%	Mean RT	CI 95%
Whole	1956.76	[1870.75, 2042.78]	1890.65	[1798.46, 1982.85]	1805.11	[1631.47, 1980.48]
Part	1749.55	[1679.99, 1819.11]	1756.46	[1678.82, 1834.10]	1669.11	[1537.56, 1800.65]

### Exploratory analyses

#### Including non-luteal cycle phase female participants

When considering all female participants including those outside the luteal cycle phase, there was no significant moderation of the part-whole effect in recognition accuracy by sex (β = 0.29, SE_β_ = 0.19, t(435.00) = 1.53, p = 0.127; 95% CI [− 0.09, 0.65]), but a significant main effect of face gender on recognition accuracy (β = − 0.27, SE_β_ = 0.14, t(435.00) = − 2.02, p = 0.044; 95% CI [− 0.53, 0.01]). When only considering luteal and non-luteal phase female participants, results show a significant interaction between cycle phase and part-whole (β = − 0.65, SE_β_ = 0.30, t(213.00) = − 2.14, p = 0.034; 95% CI [− 1.24, − 0.03]) as well as cycle phase and face gender on recognition accuracy (β = − 0.81, SE_β_ = 0.30, t(213.00) = − 2.66, p = 0.009; 95% CI [− 1.42, − 0.20]). While for luteal phase female participants we obtained no significant effect of part-whole but a significant effect of face gender (see 3.2.), just like male participants, non-luteal phase female participants showed a significant effect of part-whole (β = 0.75, SE_β_ = 0.25, t(63.00) = 3.02, p = 0.004; 95% CI [0.26, 1.24]), but no significant effect of face gender (β = 0.27, SE_β_ = 0.25, t(63.00) = 1.09, p = 0.281; 95% CI [− 0.21, 0.77]).

#### Sex hormonal modulation of holistic face processing

When adding progesterone, estradiol or testosterone to the baseline model, respectively, we did not obtain any statistically significant interaction effects with sex on the part-whole effect, leaving no indication for moderating effects of sex hormones.

For all mediation analyses, unstandardized indirect effects were computed for each of 1,000 bootstrapped samples, and the 95% confidence interval was computed by determining the indirect effects at the 2.5th and 97.5th percentiles.

**Fig. 4:**
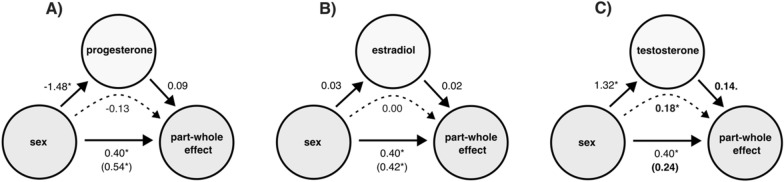
Mediation of the relation between sex and part-whole effect through **A** progesterone, **B** estradiol and **C** testosterone. Dotted lines indicate indirect effects. Numbers in brackets represent controlled direct beta effects. Asterisks indicate statistically significant effects (p < 0.05). The effect of sex was partially mediated by testosterone, whereas no evidence for mediation by progesterone or estradiol emerged

As illustrated in Fig. 4, there was a significant main effect of sex on the part-whole effect (β = 0.40, SE_β_ = 0.18, t(245.63) = 2.24, p = 0.026; 95% CI [0.04, 0.76]). As illustrated in Fig. 4. A, there was a significant main effect of sex on progesterone (β = − 1.48, SEβ = 0.12, t(122) = − 11.87, p < 0.001; 95% CI [− 1.61, − 1.07]) but no significant main effect of progesterone on the part-whole effect (β = 0.09, SE_β_ = 0.08, t(120.99) = 1.07, p = 0.285; 95% CI [− 0.08, 0.26]). The indirect effect (− 1.48)*(0.09) = − 0.13 [− 0.35, 0.10] was not statistically significant (p > 0.05; CI [− 0.03, 0.14]).

As illustrated in Fig. 4B, there was neither a significant main effect of sex on estradiol (β = 0.03, SEβ = 0.19, t(120) = 0.14, p = 0.890; 95% CI [− 0.34, 0.39]), nor a significant main effect of estradiol on the part-whole effect (β = 0.02, SEβ = 0.06, t(119) = 0.31, p = 0.759, 95% CI [− 0.11, 0.15]). The indirect effect (0.03)*(0.02) = 0.00 [− 0.01, 0.01] was not statistically significant (p > 0.05; 95% CI [− 0.05, 0.07]).

As illustrated in Fig. 4C, there was a significant main effect of sex on testosterone (β = 1.32, SE_β_ = 0.14, t(121) = 9.46, p < 0.001; 95% CI [0.51, 1.50]) and a moderate, non-significant main effect of testosterone on the part-whole effect (β = 0.14, SE_β_ = 0.08, t(120) = 1.74, p = 0.085; 95% CI [− 0.01, 0.29]). Furthermore, when adding testosterone to the original model, the direct effect of sex on the part-whole effect was reduced and no longer significant (β = 0.24, SE_β_ = 0.20, t(228.31) = 1.16, p = 0.247; 95% CI [− 0.17, 0.65]). The indirect effect (1.32)*(0.14) = 0.18 [0.01, 0.34] was statistically significant (p < 0.05). However, the Bayesian 95% credible interval [− 0.03, 0.14] suggests some uncertainty about the precision of the effect, indicating the need for cautious interpretation. Overall, 45% of the sex differences in the part-whole effect were accounted for by testosterone.

#### Eye fixations

During the 54 whole face recognition trials, male participants exhibited a significantly higher number of fixations on areas of interest (AOI) than luteal phase female participants (Table 6). When further looking at sex differences for each AOI independently, analysis yielded a significantly higher number of fixations on the right eye and the central region between both eyes in male compared to luteal phase female participants (Table 6). However, there were no sex differences in fixations for the mouth, left eye or nose region. When controlling for reaction time, there was only a significant difference in the number of right eye fixations (Table 6).

**Table 6:** Average absolute number of fixations (M), standard deviation (SD), t-value (t), degrees of freedom (df), p-value (p) and bayes factor (bF) per trial and controlled for reaction times (in brackets) for areas of interest (AOI) left eye, right eye, center, nose and mouth

	Male		Female luteal		t-test			
AOI	M	SD	M	SD	t	df	p	bF
All	1.31 (0.59)	0.60 (0.14)	1.08 (0.54)	0.36 (0.10)	− 2.49 (− 2.46)	109.81 (113)	0.014* (0.016*)	2.11 (2.22)
Center	1.29 (0.64)	0.64 (0.24)	1.10 (0.59)	0.38 (0.18)	− 1.99 (− 1.22)	109.94 (112.94)	0.049* (0.225)	0.90 (0.37)
Right eye	1.16 (0.56)	0.76 (0.27)	0.79 (0.42)	0.47 (0.24)	− 3.24 (− 3.03)	110.74 (107.11)	0.002** (0.003**)	10.50 (9.73)
Left eye	1.15 (0.57)	0.65 (0.25)	1.07 (0.55)	0.54 (0.23)	− 0.71 (− 0.46)	109.98 (104.38)	0.477 (0.649)	0.25 (0.22)
Nose	1.63 (0.79)	0.92 (0.32)	1.40 (0.72)	0.69 (0.30)	− 1.54 (− 1.09)	112.77 (104.33)	0.126 (0.228)	0.53 (0.34)
Mouth	1.31 (0.61)	0.96 (0.34)	1.05 (0.54)	0.58 (0.25)	− 1.77 (− 1.27)	110.36 (122.88)	0.080 (0.208)	0.66 (0.38)

*Fixations were only assessed for whole face recognition trials*

## Discussion

We employed a contemporary version of the part-whole face processing task originally developed by Tanaka and Farah [39], to investigate whether established sex differences in global and detail-oriented visuospatial processing styles manifest in performance measures of holistic face processing. Assuming higher female reliance on local or detail-oriented processing styles during the luteal phase, we proposed that both sex and sex hormone levels exert influence on the part-whole effect. Specifically, we hypothesized a diminished part-whole effect in luteal phase female compared to male participants, attributed to greater female engagement in feature-based face processing.

### Sex differences in the part-whole effect

As expected, our data revealed a significantly smaller part-whole effect in luteal phase female than male participants. In particular, the part-whole effect was only significant in male participants, while a relatively higher female recognition accuracy for isolated facial features led to similar recognition accuracies for part and whole face stimuli in female participants. This finding aligns with previous studies on sex difference in holistic versus detail-oriented processing styles, highlighting a female advantage in local processing across various cognitive domains including visuospatial attention and memory (see [27] for reviews, [40]). We therefore suggest, that enhanced luteal phase sensitivity to facial features results in richer face part information within face representations. Similar to findings of advanced female object location memory on spatial maps [18], one could view the facial configuration as a structural wireframe, that is, the map, with different face parts representing objects on that map.

Overall, female observers might not process faces “less holistically” than male observers but may be more likely to include a higher amount of facial feature information in their holistic memory representations. This inclination towards a default local processing mode in luteal phase female observers is further supported by findings of our exploratory analysis, showing that results from non-luteal phase female participants closely resemble those of their male counterparts. However, these findings are based on a relatively small sample of non-luteal participants (n = 22), and further studies should include a larger non-luteal sample for validation.

In the face part condition of our study, where overall sensory information is reduced and the relevant differing face part is indicated by default, both male and female participants demonstrate faster reaction times than in whole face conditions. This effect was significantly less pronounced in female participants, where the gap between reaction times for both conditions is smaller. It is possible that during recognition, a higher availability of featural information allows female observers to make more detailed comparisons, which may lead them to spend more time focusing on face parts while at the same time facilitating quicker decisions on whole-face recognition. However, this interpretation remains highly speculative, and further research is required to gain a clearer understanding of the observed sex difference in the part-whole reaction time gap.

It should be noted that previous research on sex hormonal modulation of global and local processing styles primarily used reaction time measures, whereas the part-whole effect, indicative of holistic face processing, is typically assessed through accuracy. However, in the current experiment, recognition time may not serve as a reliable measure of holistic face processing for several reasons. First, our eye fixation data indicates that participants still look at individual face parts during trials involving whole face recognition. Therefore, in whole face recognition trials, participants must first scan the entire face to identify the differing features, whereas in face-part recognition trials, only the differing feature is presented. This difference in the amount of perceptual information inherently leads to slower reaction times (RTs) for whole faces compared to face parts. Second, all participants are provided the same duration (1000 ms) for face encoding, eliminating any sensitivity to speed differences during this decisive phase. For a more valid assessment of reaction times, future research should for instance, consider adopting or developing face processing tasks that merge the presentation and response phases into a single continuous segment within each trial, ensuring that initial exposure to the stimulus and the subsequent response are seamlessly integrated.

Regarding sex hormonal influence, sex differences in the part-whole effect appeared to be partly mediated by testosterone. More specifically and in line with earlier findings [11, 13], higher levels of testosterone, known to promote global over local visuospatial processing, were related to a stronger part-whole effect. While about 46% of the observed sex difference appeared to be mediated by testosterone levels, there is still considerable room for the influence of other genetic, social and psychological factors. In line with the psychobiosocial model of sex differences e.g. [41], interactive effects of sex hormones and gender role have previously been identified as powerful mediators of sex differences in spatial navigation and mental rotations tasks [42, 43]. Since all of our participants reported matching biological sex and gender identity and since no further gender-related information is available for a more nuanced analysis, our current results may almost certainly find additional explanations beyond biological sex.

The analysis did not indicate any mediating effects of progesterone or estradiol on these sex differences. While previous findings on hormonal mediation of sex differences in visuospatial processing performance are mixed [11, 13, 44] there are several factors that may have contributed to this result. First, there were no significant differences in estradiol levels between our male and luteal female sample, effectively ruling out the possibility of estradiol-driven mediation effects. Since estradiol concentrations in saliva are typically very low, our analysis might not have had sufficient sensitivity to detect these differences. Second, the experiment only involved one test session, rendering the available hormone values less meaningful. Individual baseline sex hormone levels can vary and factors such as gene polymorphism dependent steroid receptor sensitivity [45, 46] have been reported to interact with sex hormone concentrations. Therefore, cycle phases may serve as more accurate predictors of relative hormonal influence, since they at least offer an intraindividual reference point for maximum estradiol and progesterone levels. This is a general limitation for all reported sex hormone results imposed by the current study design. Yet, the purpose of this study was to begin examining general sex differences in holistic face processing and to explore a potential relationship with sex hormone levels. It will be the task of future research to include multiple test sessions to accurately model the impact of intra-individual changes in sex hormone levels on processing performance.

### Own gender bias in face part processing

Since participants were required to decide between two highly similar faces differing in only one face part, rather than identifying whether they had previously encountered one among multiple distinctly different faces, the part-whole task substantially diverges from conventional face recognition tasks. Nonetheless, our analyses successfully replicated previous findings [4] of a significant female own-gender bias that was only present in luteal phase participants. Moreover, the female advantage in face part recognition was only observed in trials involving faces of women. This suggests that previous findings of enhanced female memory for faces of women may predominantly reflect advantages in memory for face parts rather than face configurations.

However, while a local processing advantage in female observers might explain a profound reliance on facial features in face recognition, it does not account for why this effect is absent for faces of men. A possible explanation for this discrepancy might lie in hemispheric specialization and the modulation of hemispheric communication by sex and sex hormones. More precisely, the visual system includes a right-specialized dorsal stream quickly processing coarse, low-spatial frequency information about global aspects, and a left-specialized ventral stream slowly processing rich, high-spatial frequency information about local details [47]. The resulting model of right hemispheric (RH) global and left hemispheric (LH) local specialization has received broad support from neuropsychological [48], neuroimaging [49, 50] and behavioral evidence in pattern recognition [11, 51], although some studies suggest otherwise [52, 53]. Correspondingly, advanced neuroimaging techniques like EEG and fMRI, as well as neurostimulation methods such as transcranial magnetic stimulation (TMS) have confirmed that face processing relies on a system involving both the global or holistic processing capabilities of the right hemisphere and local or featural processing expertise of the left hemisphere (see [54] for a review). More specifically, ERP and fMRI data reveal distinct lateralized activations for configural and featural face processing in the FFA [55, 56], but see [57]. In addition, TMS results revealed a significant role of right inferior frontal involvement in holistic processing, while the left middle frontal gyrus was found to be involved in analytic processing [58]. Interhemispheric communication is further believed to be influenced by organizing and activating effects of sex hormones (see [59] for a review), enhancing processing in the local-level specialized left hemisphere in female individuals during the high progesterone and luteal cycle phase [13].

A leading theory on sex hormone dependent activation or deactivation of hemispheres throughout the menstrual cycle suggests a decisive role of progesterone and estradiol dependent hemispheric decoupling [60]. Accordingly, right-hemispheric dominance for visuospatial processing introduces functional hemispheric asymmetries (FHAs) by exerting negative interhemispheric connectivity (inhibition) to the non-dominant left hemisphere via a combination of excitatory interhemispheric projections and inhibitory interneurons [60].

In such a case, local level specialized LH processing would be impaired while global level specialized RH processing can continue without restrictions.

Allopregnanolone, a neuroactive steroid metabolized from progesterone, is especially high in women during the midluteal cycle phase and is believed to enhance the inhibitory effects of GABA on excitatory transcallosal projections. This ultimately results in interhemispheric disinhibition. Estradiol, on the other hand, is assumed to excite activity in both hemispheres, which might especially facilitate processing in the inhibited non-dominant hemisphere [59, 61]. Hence, during the luteal cycle phase, both mechanisms, disinhibition and excitation, are considered to reduce hemispheric asymmetries. With LH inhibition being alleviated, the brain can take advantage of the unique specializations of both hemispheres through bilateral processing, promoting LH local processing without negatively affecting RH global processing.

Although not directly for face processing, fMRI data has already confirmed reduced global processing and reduced interhemispheric inhibition in females in the luteal compared to follicular phase during a global–local number comparison task [12] but also decreased interhemispheric connectivity in both the pre-ovulatory and luteal phase compared to menses during spatial navigation [62]. Moreover, behavioral evidence indicates diminished global precedence in naturally cycling women during the high progesterone luteal cycle phase compared to men [11, 13], women during low progesterone cycle phases and oral contraceptive users [13], who deviated from the required hormonal profile due to synthetic steroid administration. Therefore, similar to its role in promoting left-hemispheric local processing during pattern recognition, the luteal cycle phase may also enhance left-hemispheric featural face processing. However, interhemispheric decoupling represents only one of several models proposed to explain the activating effects of sex hormones on hemispheric asymmetries, as findings remain highly debated. For example, some studies report reduced FHAs exclusively during the follicular phase, while others observe the lowest asymmetry during menstruation (see [59] for a review). Additionally, the influence of sex hormones on lateralization patterns appears to be highly task-dependent, and the exact mechanisms underlying these hormonal interactions remain unclear.

Considering the aforementioned evidence, the left hemisphere has been suggested to play a key role in processing of own group faces [7, 63]. More specifically, female observers showed greater activation in the left fusiform gyrus (FFG) for female faces compared to male faces during incidental face encoding. In contrast, male BOLD responses were not significantly modulated by face gender. This enhanced left hemispheric activation in female observers was also associated with better recognition memory for faces of the same gender, underscoring the role of the left hemisphere in gender-specific differences in face recognition. Therefore, if luteal phase female participants show higher engagement in left hemispheric processing, the part-whole effect might be even smaller for faces of the observer’s own group—likely faces of their own gender—compared to those of another gender.

Yet, while this might justify a feature-based approach to processing faces more similar to one's own identity group, it does not explain the absence of the own-gender bias in male observers. It seems possible that neither mechanism alone accounts for the observed own-gender bias in luteal phase participants, but rather that a combination of own-group and cycle phase-driven increases in left-hemispheric engagement promotes featural face processing of faces of women. Moreover, the absence of an own-gender bias and reduced face part recognition in non-luteal phase participants lends further support to this hypothesis. Thus, the general lack of cycle phase control might explain inconsistent findings reported in previous studies regarding the own-gender bias in face recognition (compare 4).

### Sex differences in eye-fixation

Our exploratory analysis revealed a significantly higher number of male fixations on the right eye-region potentially linked to the female left-eye bias [64] that is also present in the current sample. Moreover, there was a significantly higher number of male fixations on the central region between the eyes that was not sustained when controlling for reaction time. The central region is not one of the varying features in our experiment and has previously been suggested as an optimal focal point for holistic face processing [65, 66], as it may be advantageous for capturing both featural and configural information. However, eye fixation patterns were assessed only for the forced-response face retrieval phase, where the absence of a time limit likely encouraged more natural fixation patterns than the constrained 1000 ms study phase, which biased eye fixations towards the task-relevant facial features. Therefore, participants may use significantly different fixation patterns during the encoding and retrieval stages of the part-whole task. Moreover, this data was only available from half of the trials, that is, only for whole face recognition trials. Consequently, the obtained fixation patterns can, if at all, only indirectly hint at possible fixation patterns that observers may use during natural face encoding and future studies would have to test the relationship between fixation patterns and recognition performance with a more targeted design.

## Conclusion

We discovered significant sex differences in the part-whole face recognition task, with female participants during the luteal cycle phase in particular showing higher response accuracies for face parts of women than male participants. This sex difference, akin to numerous previous studies involving global–local processing, was partly mediated by testosterone. Exploratory analyses further revealed a significant cycle difference in face part recognition between luteal and a small sample of non-luteal phase participants which closely resembled the discovered sex difference, but no significant differences in part-whole effects between non-luteal and male participants. Finally, the exploratory finding of significantly more frequent male eye fixations on the central face region between the eyes during face recognition could hint at a potential role of overt attentional mechanisms in the emergence of sex differences in visuospatial processing and may present an intriguing avenue for future research.

### Perspectives and significance

Our results demonstrate that sex- and menstrual cycle-related differences in global and detail-oriented processing styles observed for cognitive domains such as pattern recognition, spatial navigation or object location memory may also be relevant for complex visual stimuli like faces. Moreover, our findings suggest possible mechanisms behind established sex effects like the own-gender bias in face recognition, by approaching face processing in a more nuanced way, through separating whole and part-based face recognition. The presence of an own gender bias in face part recognition aligns well with our current understanding of functional hemispheric asymmetries in global and local processing and their modulation by sex and sex hormones. We propose that a combination of enhanced left-hemispheric processing during the luteal cycle phase and own-gender face processing might be responsible for the own-gender bias in face recognition in female observers. Future research should consider the effects of additional psychobiosocial factors on part-whole face recognition performance.

## Supplementary Information

Additional file 1.

## Data Availability

The datasets generated and/or analyzed during the current study are available in the figshare repository, https://figshare.com/s/505b7fbd104b0cfe827a.
