# Supplementary material for: Own-gender bias in facial feature recognition yields sex differences in holistic face processing
Source: Biol Sex Differ. 2025 Feb 19;16:14. doi: 10.1186/s13293-025-00695-7 (PMC11841357; doi:10.1186/s13293-025-00695-7)
Supplement: Supplementary file 1 — Additional file 1. [file 13293_2025_695_MOESM1_ESM.docx]

| **Reaction time ~ pw_condition*facegender*sex + (1\|PNr)** | | | | |  | | | | | | |
| --- | --- | --- | --- | --- | --- | --- | --- | --- | --- | --- | --- |
|  | | |  | **LMM** | | | |  | **BMLM** | | |
|  | **β** | **SE** |  | **df** | | **t** | **p** |  | **CI_low_** | **CI_up_** | **PP_b ≠ 0_** |
| (Intercept) | -0.26 | 0.12 |  | 207.23 | | -2.23 | .027 |  | -0.49 | -0.04 | 98.70% |
| sex (male) | -0.01 | 0.16 |  | 207.23 | | -0.04 | .969 |  | -0.31 | 0.30 | 52.03% |
| pw_condition (whole) | 0.33 | 0.08 |  | 435.00 | | 4.25 | < .001*** |  | 0.17 | 0.48 | 100% |
| facegender (man) | 0.11 | 0.08 |  | 435.00 | | 1.48 | .140 |  | -0.04 | 0.27 | 92.80% |
| sex : pw_condition | 0.26 | 0.11 |  | 435.00 | | 2.43 | .016* |  | 0.05 | 0.48 | 99.20% |
| sex : facegender | 0.09 | 0.11 |  | 435.00 | | 0.86 | .390 |  | -0.12 | 0.30 | 81.08% |
| pw_condition : facegender | -0.09 | 0.11 |  | 435.00 | | -0.85 | .399 |  | -0.31 | 0.12 | 79.63% |
| sex : pw_condition : facegender | -0.23 | 0.15 |  | 435.00 | | -1.47 | .141 |  | -0.54 | 0.09 | 92.50% |

**Supplementary Table 1:**  Results of Linear Mixed-Effects Models (LMM) and Bayesian Multilevel Regression Models (BMLM) for reaction times also including non-luteal phase participants in the female group. Standardized beta coefficients indicate changes in reaction times when moving from level 0 to 1 (level 1 is indicated in brackets).

| **accuracy ~ pw_condition*facegender*sex + RT + (1\|PNr)** | | | | |  | | | | | | |
| --- | --- | --- | --- | --- | --- | --- | --- | --- | --- | --- | --- |
|  | | |  | **LMM** | | | |  | **BMLM** | | |
|  | **β** | **SE** |  | **df** | | **t** | **p** |  | **CI_low_** | **CI_up_** | **PP_b ≠ 0_** |
| Intercept | 0.28 | 0.24 |  | 305.30 | | 1.17 | .242 |  | -0.19 | 0.75 | 87% |
| sex (male) | -0.66 | 0.17 |  | 398.80 | | -3.81 | <.001*** |  | -1.00 | -0.31 | 100% |
| pw_condition (whole) | 0.14 | 0.16 |  | 373.50 | | 0.85 | .396 |  | -0.18 | 0.46 | 79% |
| facegender (man) | -0.51 | 0.16 |  | 368.80 | | -3.18 | .002** |  | -0.83 | -0.20 | 100% |
| RT | 0.00 | 0.00 |  | 265.80 | | -0.26 | .798 |  | -0.00 | 0.00 | 59% |
| sex : pw_condition | 0.49 | 0.21 |  | 371.40 | | 2.33 | .021* |  | 0.09 | 0.89 | 99% |
| sex : facegender | 0.57 | 0.21 |  | 369.90 | | 2.74 | .007** |  | 0.16 | 0.99 | 100% |
| pw_condition : facegender | 0.46 | 0.23 |  | 368.80 | | 2.01 | .045* |  | 0.03 | 0.92 | 98% |
| sex : pw_condition : facegender | -0.50 | 0.30 |  | 370.00 | | -1.69 | .092 |  | -1.09 | 0.09 | 96% |

**Supplementary Table 2:**  Results of Linear Mixed-Effects Models (LMM) and Bayesian Multilevel Regression Models (BMLM) for accuracy with RT as additional covariate. Standardized beta coefficients indicate changes in accuracy when moving from level 0 to 1 (level 1 is indicated in brackets).

**Correlations between accuracy and reaction times**

|  |  | **all** | |  | | **female luteal** | | |  | | **male** | | |
| --- | --- | --- | --- | --- | --- | --- | --- | --- | --- | --- | --- | --- | --- |
|  |  | part | whole |  | part | | whole |  | | part | | whole |  |
| corr (r) |  | 0.02  (-0.02) | -0.03  (-0.05) |  | -0.03  (-0.05) | | 0.00  (-0.00) |  | | 0.08 (0.05) | | -0.03  (-0.07) |  |
| p-value |  | .834 (.787) | .746  (.543) |  | .820 (.633) | | .983  (.969) |  | | .500 (.694) | | .816  (.550) |  |

**Supplementary Table 3:** Correlations between response accuracy and reaction times (RT) for all RTs and only RTs of correct trials (in brackets) .


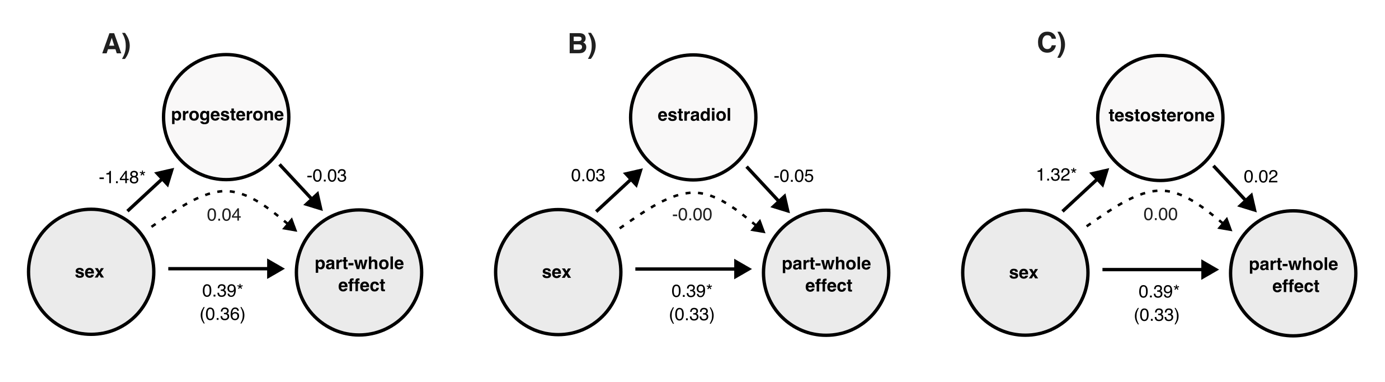


***Supplementary Figure 1:*** *Mediation of the relation between sex and reaction time part-whole effect through A) progesterone, B) estradiol and C) testosterone. Dotted lines indicate indirect effects. Numbers in brackets represent controlled direct beta effects. Asterisks indicate statistically significant effects (p < .05). The effect of sex was not mediated by sex hormones.*

| Counting Method | Days Before Onset  Of Next Menses | Progesterone (pg/ml) | Estradiol (pg/ml) | Assigned Cycle Phase |
| --- | --- | --- | --- | --- |
|  |  |  |  |  |
| acb | 11 - 3 | > 40.00 | - | luteal |
| acb | 11 - 3 | ≤ 40.00 | - | anovulatory |
| acb | 14 - 12 | - | ≤ 1.00 | anovulatory |
| ecb | 14 - 1 | > 40.00 | - | luteal |
| ecb | 11 - 6 | ≤ 40.00 | > 1.00 | non-luteal |
| ecb | 11 - 6 | ≤ 40.00 | ≤ 1.00 | anovulatory |
| ecb | > 11 | ≤ 40.00 | - | non-luteal |

**Supplementary Table 4:** Cycle phases were assigned based on either the actual cycle back (acb) or estimated cycle back (ecb) counting method. The acb method was used when the start date of menses after participation was available, while ecb was based on an estimate using the mean cycle length of the three previous cycles and the start date of menses prior to participation. When both methods were available, acb was always prioritized over ecb. (-) indicates that the criterion was not used for the respective assignment. Progesterone cutoff for the luteal cycle phase was set to 40.00 pg/ml (compare Harris et al., 2019)

Harris, T., Scheuringer, A. & Pletzer, B. Perspective and strategy interactively modulate sex differences in a 3D navigation task. Biol Sex Differ 10, 17 (2019). https://doi.org/10.1186/s13293-019-0232-z
